# Identification of Growth Phases and Influencing Factors in Cultivations with AGE1.HN Cells Using Set-Based Methods

**DOI:** 10.1371/journal.pone.0068124

**Published:** 2013-08-02

**Authors:** Steffen Borchers, Susann Freund, Alexander Rath, Stefan Streif, Udo Reichl, Rolf Findeisen

**Affiliations:** 1 Institute for Systems Theory and Automatic Control, Otto-von-Guericke University, Magdeburg, Germany; 2 International Max Planck Research School, Magdeburg, Germany; 3 Max Planck Institute for Dynamics of Complex Technical Systems, Magdeburg, Germany; 4 Institute of Process Engineering, Otto-von-Guericke University, Magdeburg, Germany; Glasgow University, United Kingdom

## Abstract

Production of bio-pharmaceuticals in cell culture, such as mammalian cells, is challenging. Mathematical models can provide support to the analysis, optimization, and the operation of production processes. In particular, unstructured models are suited for these purposes, since they can be tailored to particular process conditions. To this end, growth phases and the most relevant factors influencing cell growth and product formation have to be identified. Due to noisy and erroneous experimental data, unknown kinetic parameters, and the large number of combinations of influencing factors, currently there are only limited structured approaches to tackle these issues. We outline a structured set-based approach to identify different growth phases and the factors influencing cell growth and metabolism. To this end, measurement uncertainties are taken explicitly into account to bound the time-dependent specific growth rate based on the observed increase of the cell concentration. Based on the bounds on the specific growth rate, we can identify qualitatively different growth phases and (in-)validate hypotheses on the factors influencing cell growth and metabolism. We apply the approach to a mammalian suspension cell line (AGE1.HN). We show that growth in batch culture can be divided into two main growth phases. The initial phase is characterized by exponential growth dynamics, which can be described consistently by a relatively simple unstructured and segregated model. The subsequent phase is characterized by a decrease in the specific growth rate, which, as shown, results from substrate limitation and the pH of the medium. An extended model is provided which describes the observed dynamics of cell growth and main metabolites, and the corresponding kinetic parameters as well as their confidence intervals are estimated. The study is complemented by an uncertainty and outlier analysis. Overall, we demonstrate utility of set-based methods for analyzing cell growth and metabolism under conditions of uncertainty.

## Introduction

Production of bio-pharmaceuticals in cell culture is frequently described by unstructured and segregated models. Although the compartmental structure of cells and the underlying metabolic pathways are not taken into account explicitly, these models can provide a sound mechanistic description of the considered process, used e.g. for model-based experimental design, process optimization, or controller synthesis. A main advantage of these models is that they can be tailored to particular growth phases and process conditions. This is important since cell growth and product formation depend on a variety of factors, e.g. the availability of substrates, inhibitors, or changes in the cultivation conditions (e.g. oxygen, temperature, pH [1], [2]). However, within a particular experimental setting, only some of these factors actually contribute to the observed cell dynamics. To obtain a concise model of the process, it is necessary to identify apparent phases and the main influencing factors of cell growth and product formation.

Identifying concisely the most relevant factors influencing cell growth and product formation with respect to experimental data is however very challenging. This is, first, because the kinetics and the kinetic parameters are often unknown since they are highly cell specific and may even vary among isogenic populations [3]. Hence, the kinetic parameters have to be identified de novo for each cell line and in many cases also for each batch run. Second, available measurement data is in general uncertain, and the errors are frequently non-homogeneous, outliers may corrupt the data, and the magnitude of uncertainty is typically significant. The uncertainty affects the attainable precision of the parameter estimates, which has to be determined; and if outliers are not taken into account, this can lead to biased parameter estimates or to falsely reject hypotheses, see e.g. [4]. Third, in many situations the observed dynamics arises from the combination of several factors influencing cell growth and metabolism. This increases the complexity of the models which describe this behavior and thus complicates testing, parameter estimation and analysis, see e.g. [1], [2]. Finally, a rigorous criterion is required for testing and, if possible, rejecting hypotheses considering uncertain data.

So far, there only exist approaches to address some of the challenges mentioned above. For example, the most frequently considered approach to the hypotheses testing and parameter estimation problem is to infer the optimal parameters from the available experimental data, see e.g. [5], [6] for a comprehensive overview. To this end, an optimization problem is constructed where the model parameters are optimized considering e.g. the ordinary least squares or an appropriate information criterion in case of model selection, see e.g. [7] and references therein. Solving such optimization problems however can be difficult, because they are non-convex in general due to nonlinear system equations, see e.g. [8]. By employing a stochastic strategy (sampling, grid) for choosing initial parameters and conditions to achieve some desired global property of the solution, this limitation can be partially overcome, e.g. Monte Carlo based approaches such as simulated annealing [9] and multiple-shooting [10], or evolutionary algorithms [11], see for an in depth review [12]. Though, for a comprehensive and conclusive evaluation of options for process design and optimization, the optimal kinetic parameters do not provide sufficient information, in particular if the uncertainties are significant. Evaluating the precision of the parameters however can be very challenging for nonlinear system and non-homogeneous uncertainties, see e.g. [5], [13], [14]. So far, re-sampling techniques have been considered for this purpose, e.g. bootstrap, jackknife, and Monte-Carlo statistical methods, see [15]–[17] and references therein. These approaches are also frequently considered for uncertainty and sensitivity analysis, although they are typically limited to systems with few unknown parameters and uncertain initial conditions, see [18].

In this paper, we present a structured approach to identify the phases and the main influencing factors of cell growth and metabolism. The approach is based on a recently developed set-based method for invalidation and estimation, which is applicable to nonlinear dynamic systems and uncertain data. It builds on a semidefinite programming relaxation and efficient outer-bounding techniques [19]–[21], and is supported by the ADMIT toolbox [22]. Advantageously, the set-based method provides rigorous certificates of infeasibility used for falsification of model hypotheses, and guaranteed set-valued estimates used to determine the confidence intervals and the optimal values of the parameters and states. The set-based method for falsification and estimation is tailored here for characterizing the cell growth process, and extended to detect outliers in the data, to determine the parameter sensitivities, and to study robustness properties of the proposed models.

Particularly, we investigated the suspension growth of the human cell line AGE1.HN [23] in bioreactor and shaker flask in serum-free medium. Besides cell concentrations, the uptake of glucose and glutamine as well as the release of ammonia and lactate were measured. We distinguished two apparent cell growth phases by outer-bounding the specific growth rate as a function of time considering the observed increase of viable cell concentration. The first phase is characterized by a maximum and constant specific growth rate. This phase is described consistently by a relatively simple segregated model including the main metabolites and the dynamics of viable and dead cells. The second phase is characterized by a declining specific growth rate until growth completely ceases, where glucose limitation and the pH of the medium are the governing mechanisms for the decline of the specific growth rate in both cultivation systems.

The overall aim of this work was to obtain conclusive answers about the phases and influencing factors of AGE1.HN cell growth and metabolism. This aim was achieved by using and extending a set-based method. The structure of the paper is as follows: We first describe measurement uncertainties based on an assay validation, and introduce the most relevant aspects of the considered set-based approach. We then identify two different growth phases for AGE1.HN cells in our batch experiments. Subsequently, the growth phases are analyzed in detail, and the main influencing factors of AGE1.HN cell growth are identified. Finally, we discuss briefly the design of complementary experiments and conclude the paper.

## Materials and Methods

The methods used in this study are based on the set-based estimation and analysis framework outlined in [20], [21]. We here focus on a conceptual description of the framework and its premises, particularly how to obtain an appropriate uncertainty description of the available data. Details about the relaxation step are provided in Text S1. The study is made available for download for the ADMIT toolbox [22] (File S1). A complementary classical sensitivity and outlier analysis is provided in the Text S3.

### Model and data uncertainty description

We consider the reaction environment well mixed, and can neglect inherent stochastic effects because the amount of initial cells and substrate molecules is very large as well as the occurring reactions are sufficiently fast. Therefore, the cell growth process studied here can be described by ordinary differential equations. In general, the system's equations typically derive from balancing, considering a set of relevant compounds 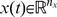 (here concentrations of the cells and extra-cellular metabolites) and their reactions. Frequently used kinetics for unstructured models are mass action, Monod, or Hill kinetics with constant reaction parameters 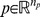, see e.g. [24]–[26]. The polynomial model equations (for 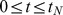) are then given by
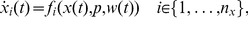
(1)where 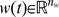 denotes inputs or time-variant parameters for sake of generality. If the initial conditions and the model parameters are known precisely, such a model allows us to make predictions about the outcome of an experiment by numerical simulation. Though, if the kinetics, the parameters, or the initial conditions are unknown, they have to be identified from experimental data beforehand. In the present case, batch culture experiments (refer Cultivations) have been performed for this purpose. We denote the observations of the extra-cellular metabolites and cell concentrations by



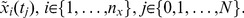
(2)For this study, we used an assay validation, see e.g. [27], [28], to quantify the measurement uncertainties. Particularly, we evaluated if the variances, for each extra-cellular metabolite and the cell concentrations, were homogeneously distributed or not using the F-test. Subsequently, the standard deviation or the relative standard deviation of the method respectively was used to determine the respective 1-sigma confidence intervals used thereafter as hard uncertainty bounds as follows:

#### Homogeneous (absolute) errors

In case variances are homogeneously distributed (according to the F-test), we consider the standard deviation of the method 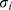 regarding a calibration function of first order (two degrees of freedom) to derive the 1-sigma confidence intervals, see e.g. [29]. The 1-sigma confidence interval is given by 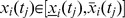, where
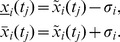
(3)


#### Non-homogeneous (relative) errors

In case the variances are non-homogeneously distributed (according to the F-test), we consider the relative standard deviation of the method 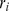 (variation coefficient), see [29] for details. The confidence intervals are then described by 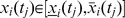, where
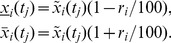
(4)


We furthermore have to take into account that the compounds are only detectable above a certain threshold. We denote the **limit of detection (LOD)** 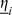 as the lowest level at which a compound concentration can be detected. The detection threshold is taken into account by
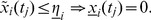
(5)


Note that, for sake of simplicity of notation, we collect the uncertainty bounds for all measured compounds by sets by
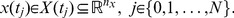
(6)


The sets 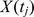 can be conveniently expressed by polytopes, which is used in our algorithms [22].

#### A priori knowledge

Very frequently, knowledge about feasible values of the states or the parameters is available independent from the experiments. Such information is very important for testing and estimation, particularly if experimental data is sparse.

Typically, the system's states can be constrained by first principles such as conservation relations (mass, momentum, energy,...) or symmetry properties, see e.g. [30]. In addition, the possible parameter values may be constrained by previous experiments. We denote the available a priori knowledge by
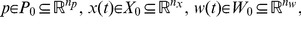
(7)where 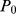, 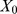, and 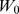 are (polytopic) bounding sets of the parameters, states, and time-variant parameters respectively.

#### Qualitative data

Qualitative information about the process can also be relevant for estimation purposes. Exemplary, the concentration of an extra-cellular metabolite is always non-negative, and substrates (glucose and glutamine) may only be consumed; hence, their concentration does not increase during the process. The by-products lactate and ammonia are released only, and thus their concentration does not decrease during the process. Such a qualitative information, exemplary the non-decreasing dynamics, can be taken into account by constraints of the form
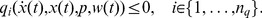
(8)


### Invalidation, estimation, and analysis

The invalidation and estimation problem are tackled in our approach by combining the model equations and the data within the following optimization problem
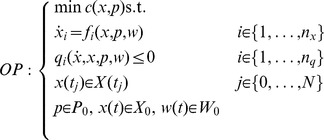
(9)Hereby, 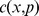 denotes a (polynomial) objective function. Solutions of above problem provide the desired results. We denote the solution 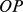 by 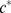.

In particular, if above 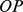 has no solution for any choice of 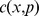, then by construction, the model is *inconsistent* with the data, i.e. there exists no state trajectory which connects all measurements. This way, a model hypothesis is falsified. To obtain the precision of a the parameter, we determine its bounding set. To this end, consider the objective 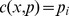 of 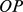, and the respective solution 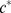. This solution defines by construction a lower bound of the parameter, 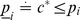. To obtain an upper bound, we consider 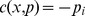, and respectively 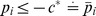. The interval 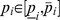 is denoted the parameter uncertainty interval. Analogously, state uncertainty intervals can be obtained by solving 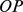. Finally, for optimization purposes, the weighted sum of least squares can be considered, i.e.
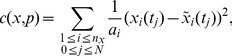
(10)where 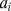 denotes the weighting factors.

Due to nonlinear model equations, the 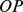 is typically non-convex and may be ill-posed, see e.g. [31]. Thus, finding the desired optimum or showing that no solution exists, is difficult in general. To obtain the desired results, we consider first a discretization of the polynomial ODEs. In a next step, the non-convex 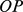 is relaxed into a semidefinite, and hence convex, optimization problem (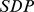), as outlined for the present settings in Text S1. Note that, 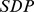
s can be solved in polynomial time with arbitrary precision [32], [33], e.g. via primal-dual interior-point methods. Note also that relaxation tightness is very difficult to quantify in general besides some particular problem classes, see e.g. [34]. However, the relaxation error can be decreased e.g. by taking additional constraints into account, see e.g. [35], or by applying partitioning strategies (e.g. bisectioning). Performance can be increased by relaxing the 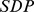 further into an linear optimization problem following the relaxation hierarchy proposed by [35].

The most important relation of the relaxed (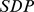) and the original optimization problem (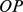) is that any solution of the original is also a solution of the relaxed one. This implies that no feasible solutions are missed. Furthermore, if both problems are feasible, then the optimum of 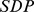 is a lower bound for the global minimum of 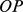. Infeasibility *certificates* or lower bounds are obtained via the dual problem of 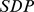, see e.g. [36]. An infeasibility certificate provides a rigorous falsification criterion, e.g. for testing model hypotheses of the specific growth rate:

#### Invalidation

*If the dual 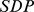 is unbounded, then 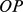 has no feasible solution. Hence, the model hypothesis is inconsistent with the data and rejected.*

Dual feasible solutions are used for estimation, i.e. to determine the uncertainty intervals of the parameters and the states.

#### Estimation

*The parameter uncertainty interval 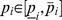 (state uncertainty interval 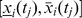 respectively) is obtained from two feasible solutions of the dual 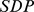.*

We focus on estimating the parameter and state 1-sigma (68.3%) confidence intervals, although more general set-valued estimates can be obtained if required. For obtaining an optimal estimate, a branch-and-bound algorithm is considered, see Text S1.

*Remark*: The computational complexity is as follows: For invalidating one model hypothesis, one SDP had to be solved. To obtain a parameter confidence interval, two SDPs had to be solved. For branch-and-bound parameter optimization, 64 SDPs had to be solved for each parameter. On a standard 2.4 GHz Intel desktop with 4 GB RAM using the ADMIT toolbox [22], the underlying SDPs for the exponential growth phase were solved in approx. 10 s, and for the complete time course of the measurements were solved in approx. 60 s.

#### Parameter sensitivity

The ‘spread’ of a parameter uncertainty interval indicates the range of possible variations of that parameter. Because any parameter value outside the interval leads to invalidity of the model by construction, the larger the interval, the less important is such a parameter variation regarding invalidity; vice versa, a parameter is sensitive, if already small variations leads to rejection of the model (hypothesis). To measure this sensitivity of the parameters, we evaluate the largest possible variation of a parameter 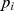 which does not lead to rejection; the sensitivity is derived from the 1-sigma confidence limits of the parameters, and is given by
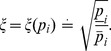
(11)


By definition, we have 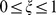. The closer the sensitivity index 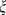 of a parameter 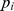 is to 1, the more sensitive is the parameter (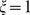 means that already a small variation of the parameter leads to rejection of the model). Sensitive parameters have sensitivity indices between 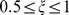, i.e. less than a 4-fold variation of the nominal parameter is possible. Values between 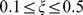 indicate less sensitive, and 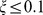 insensitive parameters (i.e. more than 100-fold variation is possible). The proposed indices are compared with classical local and Latin hypercube based global parameter sensitivity analysis in the Text S3.

#### Uncertainty analysis

Uncertainty analysis deals with the issue of investigating how uncertainty in initial conditions and parameters propagates to the model outputs, see e.g. [18]. This is of particular relevance because investigating only the nominal system's behavior (e.g. regarding fixed parameters and initial condition) does not provide insight into qualitative features such as robustness or sensitivity of the model. To evaluate the model's dynamics under uncertainties, we perform a reachability analysis, i.e. we outer-bound the feasible system's states given uncertain initial states and parametric uncertainties, i.e. the beforehand estimated parameter confidence intervals.

#### Outlier analysis

Outliers often arise due to faults, changes in systems environment, human or instrument error, or simply through natural deviations in populations, see e.g. [37]. As pointed out by [38], outliers may contain valuable information, can however lead to reject falsely a hypothesis or biased parameter estimates. Therefore it is important to identify outliers prior to modeling. Their detection is achieved here as follows: We introduce initially an additional pessimism of 10% (relative error) for dead cells (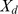) and 5% for the other five state variables. Based on this pessimism, we can estimate the model parameters and perform a reachability analysis as described before. By comparison of the so obtained reachable state sets with the measurement data, outliers can be detected and removed; Finally, the pessimism is reduced and the procedure is repeated until all outliers are detected and no pessimism is required any more.

Note that the proposed outlier detection approach is *model-generic*; a consequence is that outliers are classified regarding a particular model (hypothesis). The main advantage of this approach is that even without removing all outliers, the model can be analyzed and the parameters can be refined. In turn, the estimates improve when outliers are removed from the data. The detected outliers are furthermore validated considering a Grubbs test and the method of least trimmed squares in the Text S3. Thus, the proposed outlier analysis allows us to differentiate the rigorous invalidation criterion. We now allow for the possibility that the measurement data can be corrupted by some (few) outlying observations. However, if e.g. consecutive outliers are detected, a careful investigation is required; consecutive outliers in general constitute a rejection criterion and motivate model changes.

*Remark*: The computational complexity for the outlier detection phase is as follows. A reachability analysis (state estimation) has to be performed for each state at each time step (2.5 h). For the complete time course of the measurements (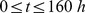), thus 768 SDPs had to be solved. Additionally, the 8 parameters were evaluated (16 SDPs). We iterated 3 times the overall procedure, summing up to approx. 2400 SDP evaluations (1200 SDP evaluations for the exponential growth phase). After identification of the outliers, the additional pessimism was dropped, and the overall procedure once more employed.

### Cultivations

The batch experiments were carried out with AGE1.HN cells [23] provided by ProBioGen AG, Berlin. This novel human cell line was immortalized by insertion of particular genes [39] and was then adapted to grow in suspension in a chemically defined medium (42-Max-UB, TeutoCell AG, Bielefeld, Germany). The medium was supplemented with 30 mM glucose and 5 mM glutamine. A stirred tank reactor (DasGip, Jülich, Germany) with a working volume of 500 ml was used to perform the bioreactor experiments. Temperature and dissolved oxygen concentration were controlled at 37°C and 40% 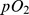 respectively. The pH value was controlled to 7.15. In contrast to this, in the shaker experiments (baffled shaker flasks, corning, working volume 150 ml) the initial pH value was 7.27, but allowed to decrease during the process. The temperature in the incubator was set to 37°C and the CO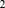 concentration was about 5%. Cell numbers were measured via automatic cell counting using the Vi-CELL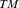 XR (Beckmann Coulter, Brea (CA), USA). This cell counter discriminates dead and viable cells using the trypan blue method. Concentrations of main metabolites were determined enzymatically with the bioprofile 100plus (Nova Biomedical, Waltham (MA), USA) [40], [41].

In the following we outline a structured approach for modeling and analyzing cell growth using above set-based methods exemplary for AGE1.HN cells. Note that the approach can be applied directly to other cell lines or process conditions.

## Results and Discussion

Growth of mammalian cells depends on various factors, essentially on the availability of the substrates glucose (Glc) and glutamine (Gln). As a by-product of Glc and Gln consumption, lactate (Lac) and ammonia (Amn) are released. Basic properties of cell growth have been described in various publications for hybridoma [42], [43], myeloma [44] and CHO cells considering unstructured models, refer also [45], [46], and metabolic shifts have been investigated for AGE1.HN cells using metabolic flux analysis [23]. In general, substrate and by-product yield factors as well as the specific growth rate strongly depend on the cell line, the used medium, and the process strategy (batch or continuous). In this work, we studied growth and metabolism of AGE1.HN cells using batch experiments in two commonly used environments, a shaker flask and a bioreactor. The experimental data is provided in the Text S2. A summary of the measurement errors obtained by assay validation is shown in Tab. 1.

**Table 1:** Statistical analysis of the measurement errors by validation assay.

	Amn	Glc	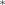 Gln	Lac	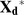	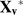
	[mM]	[mM]	[mM]	[mM]	[ 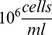 ]	[ 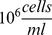 ]
LOD ( 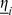 )	0.30	3.91	0.82	2.98	0.00	0.00
SD of the method (*σ_i_*)	0.03	0.39	(0.08)	0.30	(0.02)	(0.02)
% SD of the method (*r_i_*)	(2.1%)	(1.9%)	5.9%	(1.7%)	6.2%	6.2%
monotonic behavior	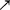	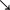	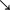	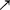	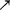	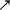

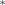
non-homogeneous variance. LOD: limit of detection. SD: standard deviation. % SD: relative standard deviation.

### Identification of growth phases

**Figure 1:**
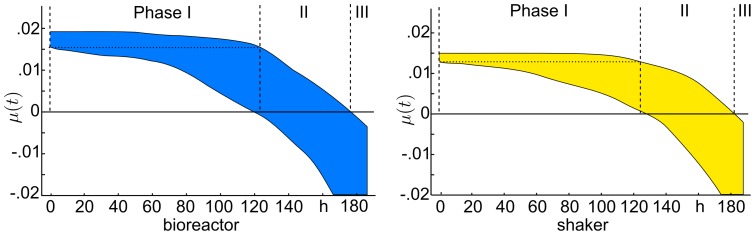
Outer bounding of the specific growth rate and identification of growth phases. Depicted is the 1-sigma confidence interval of the specific growth rate for the bioreactor (left) and the shaker (right) experiment. Phase I: exponential cell growth. Phase II: decreasing cell growth. Phase III: declining cell concentration.

Before analyzing the cell growth dynamics and the main metabolites in detail, we identified the cell growth phases based on the observed viable cell concentration 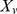 and a simple mechanistic model given by
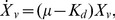
(12)where 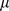 the unknown specific growth rate, 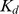 the specific cell death rate, and 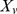 denotes the concentration of viable cells. We considered the specific cell death rate fixed (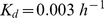, data not shown) and outer-bounded the specific growth rate considering 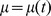 as an unknown and time-variant parameter. Particularly, we estimated the 1-sigma confidence interval of 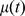 at each time sample. The results are depicted in Fig. 1.

The time-dependent specific growth rate is used subsequently to distinguish qualitatively different phases of growth of AGE1.HN cells. We characterized the first phase by the time interval where a constant specific growth rate 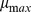 is apparent, i.e.
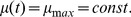
(13)Such a constant maximum specific growth rate corresponds to an exponential growth dynamic. This first phase starts at the beginning of the experiments (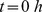), and terminates at that time point when the specific growth rate can not be considered constant any more, compare Fig. 1. The phase lasts in the bioreactor for a maximum of 125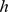, and in the shaker for a maximum of 128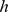. After the phase of maximum growth, the specific growth rate decreases until growth completely ceases, as shown in Fig. 1. The second phase terminates when no cell growth is observed any more, i.e. when 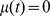. Thus, we characterized the second growth phase by the time interval where



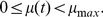
(14)For both experiments, cell growth is observed for a maximum of 180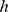. The final phase is characterized by a declining cell concentration, i.e.
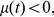
(15)observed for 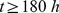.

The identification of the growth phases so far is based on the dynamics of the viable cell concentration alone. In the following, we investigated the first two indicated growth phases more comprehensively by taking the dynamics of the metabolites into account.

### Phase of exponential cell growth

To investigate the exponential growth phase, we considered a mechanistic description of the uptake of glucose (Glc) and glutamine (Gln), the release of lactate (Lac) and ammonia (Amn), as well as the dynamics of dead (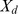) and viable cells (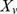), following [46] and references therein:
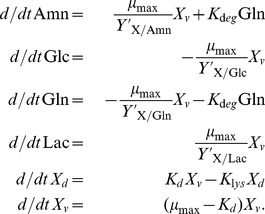
(16)


Model (16) describes cell growth under ideal conditions. It includes the uptake of Glc and Gln, the release of Lac and Amn, and the lysis of dead cells. In addition, the spontaneous degradation of Gln to Amn is taken into account (see e.g. [46] and references therein). Note that this basic model does not include feedbacks, i.e. the specific growth rate 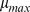 does not depend on the concentration of substrates or released products. Note also that the simple model (16) is only valid for non-negative concentrations and for low levels of accumulated by-products.

#### Parameter estimation and sensitivities

Besides the values of the parameters 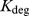 and 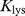, which are known from previous experiments (data not shown, see Tab. 2), the parameters of the model (16) were unknown. To estimate the four yield factors, the death rate 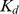, and the specific growth rate 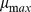, we considered the available data in Phase I, and took the 1-sigma confidence intervals of the measurements into account. As a remark, the estimation does not depend on a guess neither for the initial parameters nor the initial conditions. Instead, the range of initial parameters covers several orders of magnitudes, compare Tab. 2, and also the initial conditions were uncertain.

**Table 2:** Summary of parameters corresponding to the bioreactor and shaker flask experiment.

par.	unit	references	bioreactor			shaker		
			[ 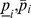 ]	*opt*	ξ	[ 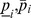 ]	*Opt*	ξ
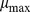	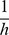	2e-2–1.3e-1	[1.54,1.91]e-2	1.90e-2	0.90	[1.26,1.48]e-2	1.44e-2	0.92
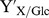	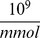	6e-2–1.7e0	[0.93,1.93]e-1	1.44e-1	0.69	[1.41,3.75]e-1	2.00e-1	0.61
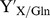	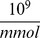	3e-2–1.6e0	[3.31,6.23]e-1	4.69e-1	0.73	[5.37,11.4]e-1	6.98e-1	0.69
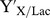	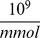	7e-2–2.5e-1	[6.20,8.28]e-2	8.22e-2	0.87	[7.58,9.60]e-2	7.76e-2	0.89
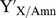	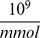	5.0e-1–2.0e0	[3.98,6.03]e-1	5.63e-1	0.81	[4.69,7.55]e-1	6.13e-1	0.79
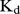	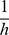	2.8e-4–3e-1	[1.66,3.45]e-3	2.66e-3	0.69	[5.09,12.4]e-4	7.20e-4	0.64
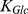	*mM*	1.5e-1–1.0e0	[0.89,2.43]	1.45	0.61	– 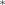		
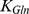	*mM*	6e-2–8.0e-1	[0.01,1.35]	0.26	0.11	[0.13,1.52]	0.49	0.29
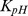	pH		– 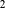			[0.51,4.91]	3.01	0.32
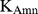	*mM*	1.0e0–2.0e1	– 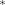			[5.16,15.8]	7.21	0.57
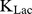	*mM*	8.0e0–1.4e2	– 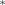			[27.7,72.9]	54.4	0.62
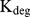	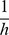	1.5e-3 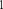	–					
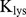	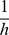	1.0e-2 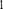	–					
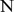	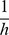	7.15e0	–					

Literature values taken from [42], [56]–[61]. 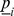 and 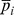 denote the lower and upper limit of the 1-sigma parameter confidence interval. 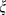 denotes the sensitivity coefficient (Eq. 11). 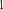
unpublished data. 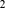
pH constant. 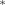
insensitive parameter.

**Figure 2:**
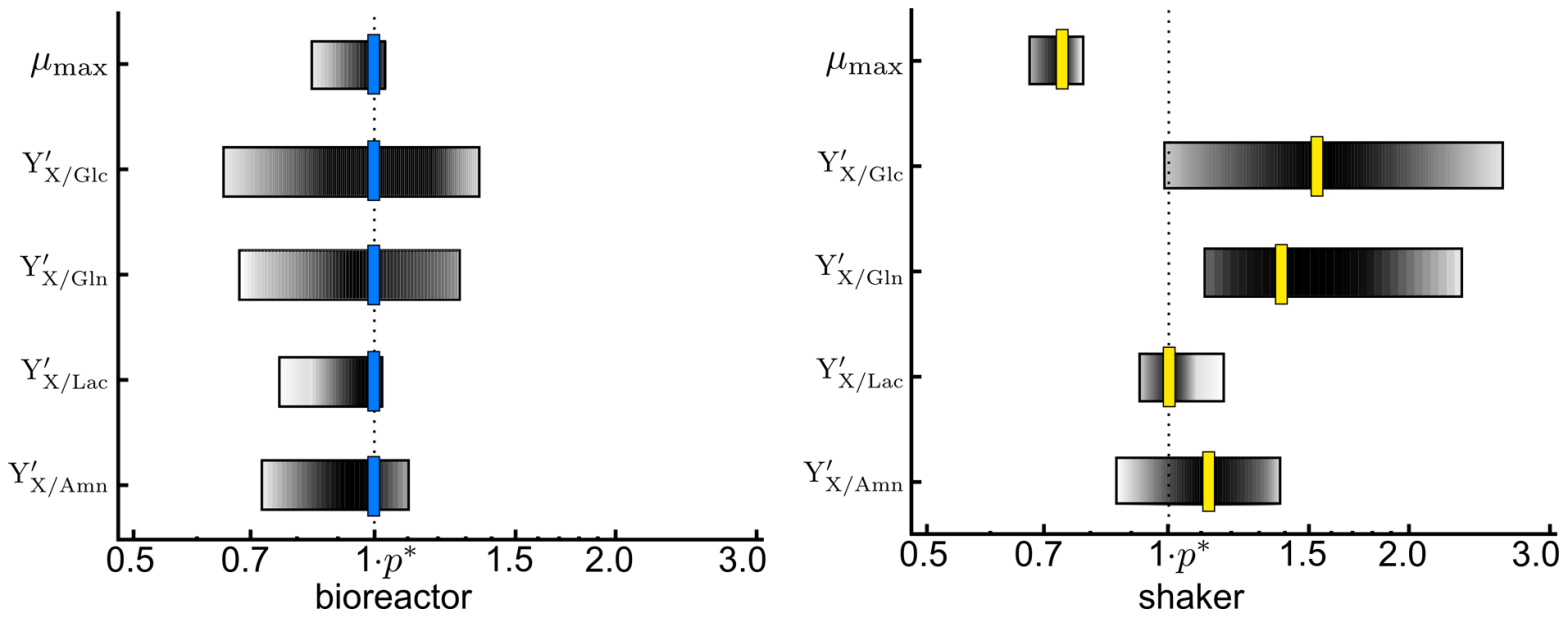
Outer bounding and optimal parameter estimation. Depicted are the parameter confidence intervals (logarithmic scale, normalized), and the optimal parameters (vertical bars) regarding the sum of least squares via branch-and-bound.

Subsequently, we determined the 1-sigma (68.3%) parameter confidence intervals and evaluated their sensitivity according to Eq. (11). The results are shown in Fig. 2 and Tab. 2. Results showed that all the unknown parameters are sensitive. Conversely, this means that the experimental data contains sufficient information for identification of the unknown parameters. We found the maximum specific growth rate 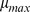 is the most sensitive parameter (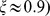; the sensitivities 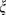 of the yield factors range from 0.6–0.9.

In a next step, we estimated the optimal parameter values regarding the least squares criterion (10) by using the proposed branch-and-bound scheme, see Text S1. The optimization results are depicted in Fig. 2. As expected, the confidence intervals were not symmetric regarding the optimal parameter values, which results from non-homogeneous errors and nonlinearity of the estimation problem.

Comparing both setups, the maximum specific growth rate is found to be larger in the bioreactor than in the shaker flask. In conclusion, the bioreactor provided more suitable growth conditions for AGE1.HN cells. Furthermore, the yield factors for the substrates, 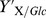 and 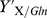, are significantly lower in the bioreactor, i.e. the substrates are utilized more efficiently in the bioreactor than in the shaker to form viable cells.

#### Uncertainty and outlier analysis

**Figure 3:**
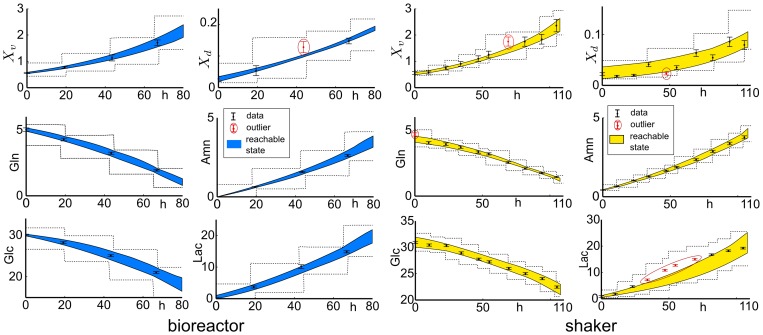
Uncertainty analysis and outlier detection (exponential growth phase). Reachable state sets are shaded, outliers are in a circle.

To evaluate the effect of uncertain parameters and to detect outliers, we estimated the reachable states of Model (16) regarding the determined parameter confidence intervals. The results are depicted in Fig. 3. The results showed that the model is rather robust with respect to parametric variations as expected, because the variations did not lead to significant or qualitatively different behavior.

Furthermore, by direct comparison of the reachable states with the measurement data, outliers were detected, see Fig. 3. Besides some lactate measurements from the shaker flask, we detected only few and isolated outliers. These isolated outliers can probably be explained from sampling or sample preparation errors, as well as the fact that we only considered the 1-sigma confidence limits of the parameters. Subsequently, we removed the outliers from the data set.

On the other hand, consecutive outliers as found for lactate in the shaker flask (see Fig. 3, right), can neither be explained by sampling nor sample preparation errors nor by statistics. Consecutive outliers typically indicate a model mismatch, i.e. a significant deviation of considered kinetics, e.g. additional metabolic pathways. Here, the mismatch might be explained by additional utilization of pyruvate. Pyruvate is present in the used medium in low concentrations, and subsequent utilization can induce an additional release of Lac at the beginning of the experiment. Because the concentrations of pyruvate were not measured for the experiment, we decided to not include the respective pathway explicitly. Further experiments which measure the concentration of pyruvate have to be considered to investigate this hypothesis in detail.

In summary, both parameter and uncertainty analysis supported the proposed model. Only isolated outliers have been detected, besides lactate dynamics in the shaker flask. The model parameters are all sensitive, and the uncertainty analysis demonstrated robustness of the proposed model with respect to parametric uncertainties.

### Phase of decreasing cell growth

We next considered the decrease in the specific growth rate with progressing time. In particular, we aimed to provide a concise model which describes consistently the observed dynamics for 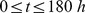, i.e. covering the complete time course of both experiments.

To this end, it was necessary to modify the structure of the basic model (16), because the model was based on the simplifying assumption that substrates were (indefinitely) available and by-product concentrations were low, which is no longer the case toward the end of the experiments. To describe a substrate uptake kinetics, we used the Monod equation (see e.g. [47], and below Eq. (18)). Substrate uptake kinetics also affects the production of Amn and Lac, because Amn is primarily produced from Gln (see [48]), and Lac from Glc, see [49]. Therefore, the production of Amn and Lac directly depends on the availability of the Gln and Glc, which had to be taken into account. The extended model considered in the remainder is given by: 
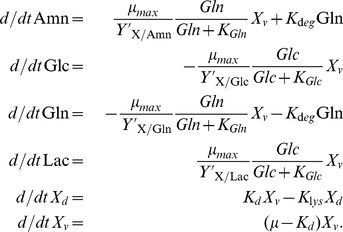
(17)


Furthermore, we had to identify the factors that explain the declining specific growth rate. In particular, we assumed that this results from negative feedbacks, e.g. substrate depletion, by-product side effects, or the pH of the medium in the shaker flask. To evaluate which of these factors actually contributed to the observed dynamics, we extended the model as described below.

First, we considered that the specific growth rate may be limited by either of the substrates Glc or Gln, e.g. [50]:
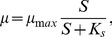
(18)where 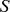 denotes the substrates concentration, and 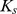 the (unknown) Monod constant. Second, accumulation of by-products may influence cell growth, i.e. Amn or Lac [50]. Such an influence can be described by a non-competitive inhibition mechanism by
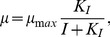
(19)where 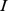 is the by-product (inhibitor) concentration, and 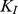 the respective (unknown) inhibition constant. Third, for bacteria and hybridoma, the influence of the pH-value on cell growth has been reported by [51] and [52], [53]. Based on their studies, the influence of the pH on cell growth can be described qualitatively by a parabola
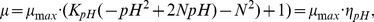
(20)where 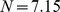 (vertex) denotes the pH value where the specific growth rate is at its maximum, and 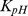 an unknown parameter. Notice that all proposed feedback hypotheses contain besides 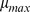 one unknown parameter. The single factor hypotheses for the specific growth rate are summarized in Tab. 3, and were analyzed hereafter. The simultaneous action of several factors is investigated later on.

**Table 3:** Specific growth rate hypotheses.

factor	hypothesis	bioreactor	shaker
Glc	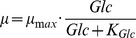	+ 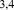	–
Gln	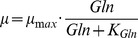	– 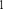	+
Lac	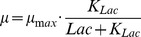	–	+
Amn	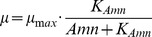	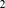 –	+
pH	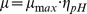	–	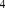 +

#### Evaluating the feedback hypotheses

**Figure 4:**
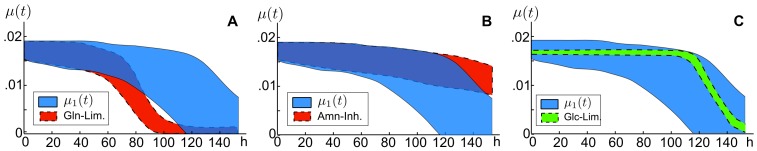
Evaluation of feedback hypotheses via invalidation. Comparison of the ‘observed’ (bioreactor) and three ‘hypothetical’ specific growth rates: Gln-limitation (A, falsified), Amn-inhibition (B, falsified), and Glc-limitation (C, validated).

For evaluation, we chose a reverse engineering approach. We already estimated the specific growth rate 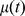 depicted in Fig. 1, which reflects the ‘observed’ cell growth dynamics. In addition, we determined the 1-sigma confidence limits for the specific growth rate according to the hypotheses listed in Tab. 3. To this end, we considered the 1-sigma confidence interval 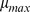 as determined before and constrained the remaining unknown parameter to the range of the reported literature values, compare Tab. 2. Thus, we obtained the ‘hypothetical’ specific growth rate, which were compared with the ‘observed’ specific growth rate for falsification purposes as shown in Fig. 4. Exemplary, Fig. 4A and Fig. 4B show the results for Gln-limitation and Amn-inhibition in the bioreactor, respectively. Because the ‘observed’ and the ‘hypothetical’ specific growth rate in both cases do not overlap at any time, we found that neither Gln-limitation nor Amn-inhibition alone explained the observed growth dynamics. On the contrary, Glc-limitation, see Fig. 4C, was found a valid hypothesis.

The results are summarized in Tab. 3. Results showed, as expected, that Glc is essential for cell growth in the bioreactor. In contrast, Gln limitation did not affected growth of AGE1.HN cells. Furthermore, we showed that the by-products Amn and Lac did not affected cell growth within the observed concentration ranges significantly (considering physiologically meaningful inhibition constants).

The situation in the shaker flask is different, because Glc is available until the end of the experiment, and shown to be not responsible for the decrease of the specific growth rate here, see Tab. 3. Instead, the decrease may be explained by by-product inhibition, the proposed pH-dependency, or Gln-limitation. Hence, without additional knowledge, the results appear to be non-conclusive for the shaker flask. However, since we showed that cell growth is not affected by Gln-limitation in the bioreactor, we could rule out this hypothesis for the shaker flask. Furthermore, since the observed concentration ranges of Amn and Lac were comparable in the bioreactor and in the shaker flask (both slightly lower in the shaker), we could rule out Amn nor Lac inhibition too. For the shaker, only the pH dependency hypothesis remained. Because it is known that the pH value decreases due to the release of the acid Lac, we finally concluded that the decrease of the specific growth rate in the shaker is the result of the acidification of the medium by Lac.

**Figure 5:**
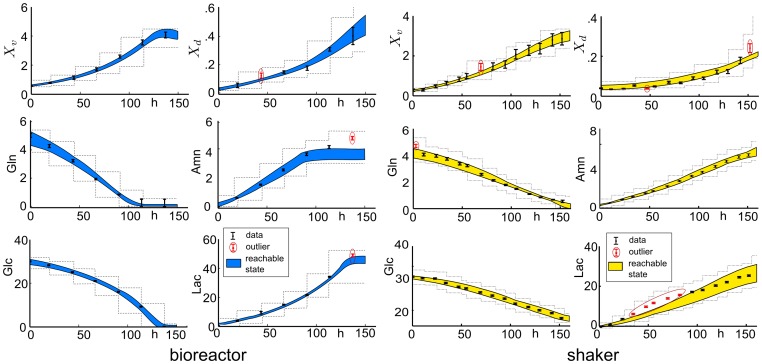
Uncertainty analysis and outlier detection. Reachable state sets are shaded, outliers are in a circle. Bioreactor: Glc-limitation, Shaker: pH-dependency.

For further analysis, we determined the unknown parameters and the corresponding confidence intervals for Glc-limitation (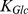) and pH-dependency (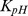), see Tab. 2. The parameters were found sensitive and in accord with the literature values. Finally, for Glc-limitation (bioreactor) and pH-dependence (shaker flask), we performed an uncertainty and outlier analysis as described before, see Fig. 5; this analysis demonstrated robustness against parametric variations, and only few (non-consecutive) additional outliers.

The considered falsification approach was then used to investigate the simultaneous action of two factors on cell growth. To this end, we considered multiplicative superposition, e.g. superposition of Glc-limitation and Amn-inhibition by
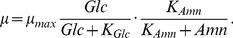
(21)


Note that it is also possible to study additive superposition of influencing factors as well as combinations that can be expressed in terms of Boolean logic.

We evaluated all possible combinations of two influencing factors for both cultivation systems. In the bioreactor, we found that only feedbacks including Glc-limitation and excluding Gln-limitation are consistent with the observations. None of the combinations could be rejected in the shaker flask. An important insight is that the feedbacks become more difficult to analyze the more influencing factors are considered. This is due to the fact that the number of unknown parameters increases, because each influencing factor introduces an additional parameter, while the available information for estimation remains the same. Here, the parameters became correlated and were therefore found less sensitive (results not shown).

Finally, to address parameter estimation in future, a design of experiments should be considered by which the possible influencing factors are investigated one by one. This avoids superposition of several influencing factors, and hence more precise parameter estimates can be expected. For evaluating by-product influences, a pulse administration during the exponential growth phase will be advantageous. This pulse should be strong enough to decrease the influence of uncertainties, within biologically meaningful limits. Similarly, the influence of pH should be studied explicitly this way.

## Conclusions

We proposed a structured approach for analyzing and characterizing cell growth and metabolism, outlined for AGE1.HN cells cultured in two commonly used environments. The key benefit of the considered set-based methods is their robust perspective onto falsification, estimation, and analysis while providing conclusive and guaranteed results. This is of particular relevance for biological and biotechnological processes, e.g. to evaluate options for process design and optimization, which frequently show persistence of the characteristic system behavior under conditions of uncertainty.

In both experiments, we identified two qualitatively different growth phases. The first phase was characterized by a constant maximum specific growth rate corresponding to exponential cell growth. We demonstrated that this phase could be described very well by a relatively simple model including the main metabolites as well as dynamics of viable and dead cells. Besides lactate dynamics for the shaker flask experiment, only few and isolated outliers were detected; the model was shown to be robust with respect to parametric uncertainties. We showed also that the bioreactor provided more suitable growth conditions than the shaker. The second phase was characterized by a declining specific growth rate. To describe the observed dynamics for the complete time course of both experiments, we extended the previous model including substrate limitations, and identified the factors which lead to the decrease of the specific growth rate. By falsification, we demonstrated that the governing mechanism for this was glucose limitation in the bioreactor, and the decrease of the pH value due to the release of lactate in the shaker. Only few additional isolated outliers were detected; overall the models were in good accord with the experimental data.

To further investigate the influence of metabolic by-products onto AGE1.HN cell growth and metabolism, additional experiments should be considered, i.e. by adding (large amounts of) by-product, or acid to evaluate the influence of pH, during the exponential growth phase. Also, the consumption of pyruvate should be investigated in future experiments. Furthermore, it would be interesting to extend the here considered mechanistic description of the viable and dead cell dynamic and main metabolite concentrations to intra-cellular metabolites. For example, extra-cellular fluxes and their error bounds can be calculated, and then be used to determine unknown intra-cellular fluxes, e.g. considering dynamic metabolic flux analysis [54], [55]. Besides, the proposed methods should be further developed to include qualitative data for estimation and analysis.

With this study, we have demonstrated that the set-based approaches are valuable tools to analyze biotechnological processes under conditions of uncertainty.

## Supporting Information

File S1 **ADMIT toolbox files.**
(ZIP)

Text S1 **Short description of the frameworks methods.**
(PDF)

Text S2 **Data tables.**
(PDF)

Text S3 **Parameter sensitivity analysis and outlier validation.**
(PDF)
