# Supplementary material for: Identification of Growth Phases and Influencing Factors in Cultivations with AGE1.HN Cells Using Set-Based Methods
Source: PLoS One. 2013 Aug 2;8(8):e68124. doi: 10.1371/journal.pone.0068124 (PMC3732265; doi:10.1371/journal.pone.0068124)
Supplement: File S1 — ADMIT toolbox files. (ZIP) [file pone.0068124.s001.zip › Supplementary_material_S2/supp_mat_S2.pdf]

Supporting Information 2

ADMIT-Toolbox files providing scripts for:

A: Bounding of the specific growth rate $\mu(t)$ The files provide the computation of the specific growth rate as a function of time. The data is provided as csv table, the file analyzeModel.m starts the script, given that the Admit toolbox is installed.

B: Parameter estimation (exponential growth phase) The files provide the setup for the parameter estimation. The data is provided as csv table, the file analyzeModel.m start the script.

C: Uncertainty analysis (exponential growth phase) The files provide the computation of the reachable state sets for the exponential growth phase. The data is provided as csv table, the file analyzeModel.m starts the script.
