# Supplementary material for: Identification of Growth Phases and Influencing Factors in Cultivations with AGE1.HN Cells Using Set-Based Methods
Source: PLoS One. 2013 Aug 2;8(8):e68124. doi: 10.1371/journal.pone.0068124 (PMC3732265; doi:10.1371/journal.pone.0068124)
Supplement: Text S1 — Short description of the frameworks methods. (PDF) [file pone.0068124.s002.pdf]

Text S1

Model discretization and sampling

To apply the set-based approach, it is necessary to transform the continuous-time ordinary differential equation system *(1)* into an equivalent system of difference equations. This is done by applying a discretization scheme and considering an appropriate sampling of the time window, i.e. $\{0, t_1, \ldots, t_N\}$. In the present case, we consider a first order approximation method known as the Euler forward method. The scheme derives from the truncation of the Tailor series considering only the first order derivative, i.e. the linear approximation (extrapolating the tangent at t_0) with

$$x(t_0 + h) \approx x(t_0) + h\dot{x}(t_0), \tag{1}$$

where h denoted the step size, and we have

$$\dot{x}(t_0) \approx \frac{x(t_0 + h) - x(t_0)}{h}. \tag{2}$$

Denoting $\dot{x}(t_0) = f(x(t), p, w(t))$, assuming $u(t)$ being constant within the time interval $t \in [t_0, t_0 + h]$ (zero-order-hold), and denoting $x_{k-1} = x(t_0)$, $u_{k-1} = u(t_0)$, $x_k = x(t_0 + h)$, we finally obtain the difference equation system

$$f_i^k(x_k, x_{k-1}, p, w_{k-1}) = 0, \quad i \in [1{:}n_x] \tag{3}$$

Here, x_k, $,w_k$ denote respectively the system states and inputs at the time index k, and $p \in \mathbb{R}^{n_p}$ the constant parameters. We furthermore choose the sampling sufficiently small to avoid discretization errors, i.e. $t_{k+1} - t_k = 2.5h$. For a comprehensive overview of higher order discretization schemes and related numerical stability issues, see e.g. [1, 2].

For simplicity of notation, we denote the collection of all variables, induced by the sampling, by

$$z \doteq (p_1, \ldots, p_{n_p}, x_0, \ldots, x_N, w_0, \ldots, w_{N-1}),$$

with $z \in \mathbb{R}^{n_z}$, $n_z = n_p + N(2 + n_x + n_w)$. The model equations can then be summarized by

$$f_i^k(z) = 0, \quad k \in [1{:}N], i \in [1{:}n_x], \tag{4}$$

with appropriate choice of z-components.

Data and uncertainty description

The a priori knowledge is modeled by polytopic sets bounding the possible variable's values. The a priori bounding sets of the parameters p, states x, and inputs w, with $0 \leqslant t \leqslant t_N$ are respectively given by:

$$D_{prior} : \begin{cases} p \in P_0 & \doteq & \{p \in \mathbb{R}^{n_p} : A_p p \leqslant a_p\}, \\ x \in X_0 & \doteq & \{x(t) \in \mathbb{R}^{n_x} : A_x x_k \leqslant a_x,\ k \in [0:N]\} \\ w \in W_0 & \doteq & \{w(t) \in \mathbb{R}^{n_w} : A_w w_k \leqslant a_w\ k \in [0:N-1]\}, \end{cases} \tag{5}$$

with known the matrix-vector pairs (A_p, a_p), (A_x, a_x), (A_w, a_w) of appropriate dimensions. The a priori data D_{prior} can then be expressed as a polytopic set

$$Z_{prior} \doteq \{z \in \mathbb{R}^{n_z} : A_{prior} z \leqslant a_{prior}\}, \tag{6}$$

where A_{prior}, a_{prior} constructed from A_p, A_x, A_w and a_p, a_x, a_w respectively.

Similarly, a measurement and its uncertainty are described by

$$D_{meas} : \{\ x(t_j) \in X(t_j) \doteq \{x \in R^{n_x} : A_{x(t_j)} x_j \leqslant a_{x(t_j)}\} \quad j \in [0:N], \tag{7}$$

with know matrix-vector pair $(A_{x(t_j)}, a_{x(t_j)})$, for all $j \in [0 : N]$. The respective polytope

$$Z_{meas} \doteq \{z \in \mathbb{R}^{n_z} : A_{meas} z \leqslant a_{meas}\}, \tag{8}$$

is constructed analogously.

Finally, the structural data, which expresses dependencies of variables,

$$D_{str} : \{ \;\; q_i(x_k, x_{k-1}, p, w_k) \leqslant 0 \quad i \in [1 : n_q] \quad k \in [0 : N]. \tag{9}$$

is expressed similarly as before by

$$Z_{str} \doteq \{z \in \mathbb{R}^{n_z} : A_{str} z \leqslant a_{str}\}. \tag{10}$$

With these preparations, we can now summarize the available data as the Cartesian product

$$Z = Z_{prior} \times Z_{meas} \times Z_{str} = \{z \in \mathbb{R}^{n_z} : A_z z \leqslant a_z\}, \tag{11}$$

where the matrix-vector pair $(A_z,\, a_z)$ is constructed from $A_{prior}, A_{meas}, A_{str}$ and $a_{prior}, a_{meas}, a_{str}$ respectively. Note that $(A_z,\, a_z)$ may contains redundant constraints, which can be detected and removed following e.g. [3].

Set of consistent solutions and optimization

All solutions of the dynamical model M (3), which are *consistent* with the data D, belong to the set $\mathcal{Z} \subset \mathbb{R}^{n_z}$ with:

$$\mathcal{Z} \doteq \left\{ z \in Z : \begin{array}{ll} f_i^k(z) = 0 & k \in [1{:}N], i \in [1{:}n_x] \\ A_z z \leqslant a_z & \end{array} \right\} \tag{12}$$

where $f^k(z)$ are the systems equations of *(1)* with an appropriate choice of z-components. We denote the respective constraint satisfaction problem (12) by $M \cap D$.

The set of consistent parameters can be seen as an n_p-dimensional axis-parallel subspace of $\mathbb{R}^{n_z}$, formalized by the projection map $f_p : \mathbb{R}^{n_z} \to \mathbb{R}^{n_p}$, i.e. $\mathcal{P} = f_p(\mathcal{Z})$. Analogously, the set of consistent states (at t_k) is given by the projection map $f_{x_k} : \mathbb{R}^{n_z} \to \mathbb{R}^{n_x}$, i.e. $\mathcal{X}(t_k) = f_{x_k}(\mathcal{Z})$.

The parameter optimization problem consists in determining the (global) optimum $c(z^*)$, i.e. to solve the polynomial optimization problem (POP)

$$POP(Z) : \left\{ \begin{array}{l} \min\limits_{z \in \mathbb{R}^{n_z}} c(z) \text{ s.t.} \\ z \in \mathcal{Z}. \end{array} \right. \tag{13}$$

SDP Relaxation

To this end, the following relaxation procedure is considered. First, we lift $POP(Z)$ to an equivalent quadratic problem by *quadrification.* Let $\mathbb{S}^n$ be the set of real symmetric $n \times n$ matrices, and

$$\langle A, B \rangle = \sum_{i,j} a_{ij} b_{ij}$$

denote the usual Frobenius product. Quadrification (see [4]) consists in deriving a monomial vector ξ for which

$$c(z) = \langle C, \xi\xi^T \rangle,\; f_i^k(z) = \langle F_i^k, \xi\xi^T \rangle,$$

for appropriate matrices $C,\ F_i^k \in \mathbb{S}^{n_\xi}$. Each *monomial* (of ξ) of degree two or more is the product of two other monomials of lower degree. As a technical requirement, we ask, without loss of generality, that $\xi_1 = 1$. The next n_z components of the vector ξ are all the components of z, i.e.,

$$(\xi_2, \ldots, \xi_{n_z+1}) = z.$$

Note we have $\xi\xi^T e_1 = \xi$, with $e_1 = (1, 0, \ldots, 0)^T \in \mathbb{R}^{n_\xi}$.

The polytopic constraint set Z bound, by construction, the z-equivalent components of ξ. The remaining components, which are monomials of degree two or higher, can be bounded directly from the data Z, particularly considering interval arithmetic. The resulting bounding constraints for all the components of ξ are expressed by

$$A_\xi \xi\xi^T e_1 = A_\xi \xi \leqslant a_\xi.$$

Finally, by replacing the product matrix $\xi\xi^T$ with a symmetric variable matrix $\Xi \in \mathbb{S}^{n_\xi}$, and considering $\Xi \succeq 0$, we obtain the convex semidefinite program:

$$SDP(Z) : \begin{cases} \min\limits_{\Xi \in \mathbb{S}^{n_\xi}} \langle C, \Xi \rangle \text{ s.t.} \\ \langle F_i^k, \Xi \rangle = 0 \qquad\qquad k \in [1{:}N], i \in [1{:}n_x] \\ A_\xi \Xi e_1 \leqslant a_\xi \\ \Xi_{11} = 1 \\ \Xi \succeq 0. \end{cases}$$

Dual certificate

Any feasible solution for the *dual problem* of a SDP provides by weak duality a lower bound to the SDP optimum [5]. Therefore, dual unboundedness provides a certificate of primal infeasibility. Moreover, if strong duality applies then the optimum of the dual and of the primal coincide, which provides a certificate of optimality.

Several SDP duals have been proposed (see [6] for a discussion of SDP duals). For simplicity, we consider the Lagrangean dual (denoted by $SDP^*(Z)$), which is itself an semidefinite program and for which strong duality holds under constraint qualification conditions (see e.g. [7]).

We denote by Ξ^* the optimal solution of the dual program $SDP^*(Z)$; by weak duality, we have that Ξ^* is a lower bound on the objective value for the $SDP(Z)$. With $z^* = f_z(\Xi^*)$, $f_z : \mathbb{S}^{n_\xi} \to \mathbb{R}^{n_z}$, we have the desired lower bound on the objective value for $POP(Z)$, i.e.

$$c(z^*) \leqslant c(z), \forall\, z \in \mathcal{Z}.$$

Furthermore, if $c(\Xi^*) \to \infty$, i.e. unbounded, we have by weak duality that $SDP(Z)$ is infeasible, and hence $POP(Z)$ infeasible.

Outer-bounding

By choosing as objective a variable of interest, e.g. $c(z) = p_i$, C_i accordingly, the respective solution of the dual SDP^*,

$$\langle C_i, \Xi^* \rangle \doteq \underline{p}_i$$

provides a lower bound on p_i. Analogously, by choosing $c(z) = -p_i$, C_i accordingly, the respective solution gives

$$-\langle C_i, \Xi^* \rangle \doteq \overline{p}_i,$$

i.e. an upper bound of the respective variable p_i. The lower and upper bounds define the (a posteriori) *uncertainty interval* $[\underline{p}_i, \overline{p}_i]$.

Partitioning

To analyze the solution sets in more detail, we consider a partitioning approach. We partition the initial bounding set into subsets, which are subsequently analyzed for infeasibility. Infeasible partitions can be discarded, and from the remaining partitions the outer-estimate is constructed. Formally, we consider a selection s of variables of interest, e.g. the parameters p. We denote the a priori feasible set by S, e.g. P_0.

We then partition S into a number of subsets, i.e. $Q_j \subseteq S$, $j \in [1\!:\!Q]$. This is achieved e.g. by some recursive scheme (e.g. binary branching) up to some desired volumetric resolution ε, e.g. the recursive bisection algorithm:

Algorithm 1 (Bisectioning(Q, ε))

1. *If $SDP^*(Z \cap Q)$ is unbounded then exit and return $\emptyset$*
2. *If $||Q|| \leqslant \varepsilon$ then exit and return Q*
3. *Partition Q into Q_1 and Q_2*
4. *Set $Q'_1 \doteq$ Bisectioning(Q_1)*
5. *Set $Q'_2 \doteq$ Bisectioning(Q_2)*
6. *Return $Q'_1 \cup Q'_2$*

The estimate is then the union of partitions Q_j for which $\langle C, \Xi^* \rangle \doteq c_j$ of $SDP^*(Z \cap Q_j)$ is bounded, i.e.

$$\mathcal{I}(\mathcal{S}) = \bigcup_{\substack{c_j < \infty,\\ 1 \leqslant j \leqslant Q}} Q_j.$$

Branch-and-bound optimization

For global optimization, we consider the objective function $c(z)$ given by the sum of least squares *(10)*. To obtain the optimal parameter values, a branch-and-bound scheme is considered. To this end, we consider a partitioning Q_j, $i \in [1 : q]$ of the initial parameter region P_0. For each partition, we evaluate the value of the sum of least squares, i.e. by solving $SDP^*(Z \cap Q_j)$, and assign to each feasible partition Q_j the lower bound. Partitions with lowest least squares are further analyzed and validated considering Monte Carlo tests. In the present case, we consider 64 partition for each parameter.

References

1. Davis P, Rabinowitz P (1975) Methods of numerical integration. Academic Press New York.
2. Ralston A, Rabinowitz P (2001) A first course in numerical analysis. Courier Dover Publications.
3. Mattheiss T (1973) An algorithm for determining irrelevant constraints and all vertices in systems of linear inequalities. Oper Res 21: 247–260.
4. Sherali H, Tuncbilek C (1997) Comparison of two Reformulation-Linearization Technique based linear programming relaxations for polynomial programming problems. J of Glob Opt 10: 381–390.
5. Boyd S, Vandenberghe L (2004) Convex optimization. Cambridge University Press, UK.

6. Ramana M, Tunçel L, Wolkowicz H (1997) Strong duality for semidefinite programming. SIAM J Optim 7: 641–662.

7. Nesterov Y, Nemirovski A (1994) Interior-point polynomial algorithms in convex Programming, volume 13. SIAM J Appl Math.
