# Supplementary material for: Identification of Growth Phases and Influencing Factors in Cultivations with AGE1.HN Cells Using Set-Based Methods
Source: PLoS One. 2013 Aug 2;8(8):e68124. doi: 10.1371/journal.pone.0068124 (PMC3732265; doi:10.1371/journal.pone.0068124)
Supplement: Text S2 — Data tables. (PDF) [file pone.0068124.s003.pdf]

Text S2

Table S1. Experimental data of bioreactor cultivation.

**time** *h*	**Amn** mM	**Glc** mM	**Gln** mM	**Lac** mM	$\mathbf{X_d}$ $10^6 \frac{\text{cells}}{\text{ml}}$	$\mathbf{X_v}$ $10^6 \frac{\text{cells}}{\text{ml}}$	**pH**
0.0	0	30.0	5.03	0	0.03	0.56	7.24
19.3	0.64	28.2	4.28	3.72	0.06	0.78	7.14
43.5	1.57	25.1	3.24	9.86	0.13	1.14	7.09
66.8	2.62	21.0	1.94	14.8	0.15	1.71	7.10
90.5	3.73	16.1	0.84	21.4	0.18	2.59	7.19
113.5	4.24	9.23	0.5	34.0	0.31	3.57	7.10
137.3	4.87	0	0.49	49.4	0.41	4.08	7.17
161.8	6.36	0	0.46	45.7	0.91	4.06	7.17
188.3	7.68	0	0.45	40.9	1.38	3.11	7.21

Table S2. Experimental data of shaker flask cultivation.

**time** *h*	**Amn** mM	**Glc** mM	**Gln** mM	**Lac** mM	$\mathbf{X_d}$ $10^6 \frac{\text{cells}}{\text{ml}}$	$\mathbf{X_v}$ $10^6 \frac{\text{cells}}{\text{ml}}$	**pH**
0.0	0.43	30.94	4.68	0.88	0.02	0.56	7.27
10.3	0.67	30.44	4.04	1.72	0.02	0.58	7.15
23.0	1.03	30.34	3.94	4.63	0.02	0.75	7.10
34.0	1.29	28.94	3.72	7.18	0.04	0.91	7.03
47.0	1.65	27.75	3.34	10.83	0.02	1.10	6.98
54.8	1.87	27.25	3.21	12.71	0.03	1.26	6.97
69.2	2.35	26.00	2.55	14.99	0.06	1.72	6.98
81.5	2.87	25.01	2.09	16.71	0.05	1.75	6.93
94.0	3.37	24.06	1.75	18.22	0.08	1.84	6.85
104.8	3.76	22.46	1.46	19.23	0.07	2.36	6.86
118.2	4.34	21.42	1.07	21.43	0.11	2.55	6.80
129.5	4.85	20.42	0.82	23.15	0.11	2.64	6.77
142.2	5.28	19.52	0.64	25.59	0.17	3.08	6.65
152.0	5.49	17.93	0.58	26.42	0.23	3.14	6.57
165.8	6.02	17.93	0.57	29.82	0.52	3.11	6.48
176.7	6.15	16.38	0.55	29.82	0.90	2.82	6.32
190.5	6.25	15.83	0.52	29.97	2.41	1.14	6.20
