# Supplementary material for: Identification of Growth Phases and Influencing Factors in Cultivations with AGE1.HN Cells Using Set-Based Methods
Source: PLoS One. 2013 Aug 2;8(8):e68124. doi: 10.1371/journal.pone.0068124 (PMC3732265; doi:10.1371/journal.pone.0068124)
Supplement: Text S3 — Parameter sensitivity analysis and outlier validation. (PDF) [file pone.0068124.s004.pdf]

Text S3

Parameter Sensitivity Analysis (PSA)

In this section, we evaluate classical parameter sensitivity coefficients and compare the results with the proposed sensitivity measure. To this end, we first consider a local parameter sensitivity analysis, and second a global parameter sensitivity approach using Latin hypercube sampling.

For simplicity, we collect the kinetic parameters as listed in Table 2 by the vector $p \in \mathbb{R}^{n_p}$, and the model states by the vector $x \in \mathbb{R}^6$. The local parameter sensitivity coefficients are derived by the forward method (see e.g. [1]), i.e. by solving the sensitivity differential equation:

$$\frac{d}{dt}\frac{\partial x(t)}{\partial p} = \frac{\partial f(x(t),p)}{\partial x(t)}\frac{\partial x(t)}{\partial p} + \frac{\partial f(x(t),p)}{\partial p}.$$

Here, $f(x,p)$ denotes the right hand side of the system given in Equations (16) (exponential growth phase, $0 \leq t \leq 80\ h$) and Equations (17) (complete time course, $0 \leq t \leq 160\ h$) with

$$\mu = \mu_{\max}\frac{Glc(t)}{Glc(t) + K_{Glc}}.$$

We denote the normalized parameter sensitivities by

$$S_{ij}(t) = \frac{\partial x_i(t)}{\partial p_j} \cdot \frac{p_j}{x_i(t)}, \tag{1}$$

and the sensitivities are ranked using the absolute infinite norm as sensitivity metric given by

$$|S_{ij}|_\infty = \max_t |S_{ij}(t)|. \tag{2}$$

The sensitivity coefficients are scaled finally by the maximum sensitivity coefficient

$$|S_{ij}|_\infty^{loc} = |S_{ij}|_\infty / \max_{\substack{1\leq i\leq n_x \\ 1\leq j\leq n_p}} |S_{ij}|_\infty. \tag{3}$$

Because the local parameter sensitivity coefficients are valid only for small perturbations of the nominal parameters, we furthermore employ a global sensitivity analysis considering a Latin hypercube sampling of initial parameter values, see e.g. [2]. To this end, we consider as parameter intervals, from which the parameter values are sampled, the obtained confidence intervals as given in Table 2. The number of samples is 10000; for each sample we calculate the sensitivity coefficients $|S_{ij}|_\infty^{loc}(k)$ according to Eq. 3 and where $1 \leq k \leq 10000$. From these samples, we infer the mean:

$$|S_{ij}|_\infty^{glob} = \frac{1}{10000}\sum_{k=1}^{10000} |S_{ij}|_\infty^{loc}(k). \tag{4}$$

Results

The sensitivity analysis results for the exponential growth phase are shown in Fig. S1, for the complete time course of the measurements in Fig. S2. Regarding the exponential growth phase, μ_{max} is the most sensitive parameter (both locally and globally), and changes in this parameter affect all the species of the system. The yield factors $\mathrm{Y}'_{\mathrm{X/Amn}}$, $\mathrm{Y}'_{\mathrm{X/Glc}}$, $\mathrm{Y}'_{\mathrm{X/Gln}}$, and $\mathrm{Y}'_{\mathrm{X/Lac}}$ are shown sensitive only with respect to Amn, Glc, Gln, and Lac respectively. K_D is particularly sensitive with respect to X_d. Regarding the complete time course of the measurements, again μ_{max} is by far the most sensitive parameter. Also the

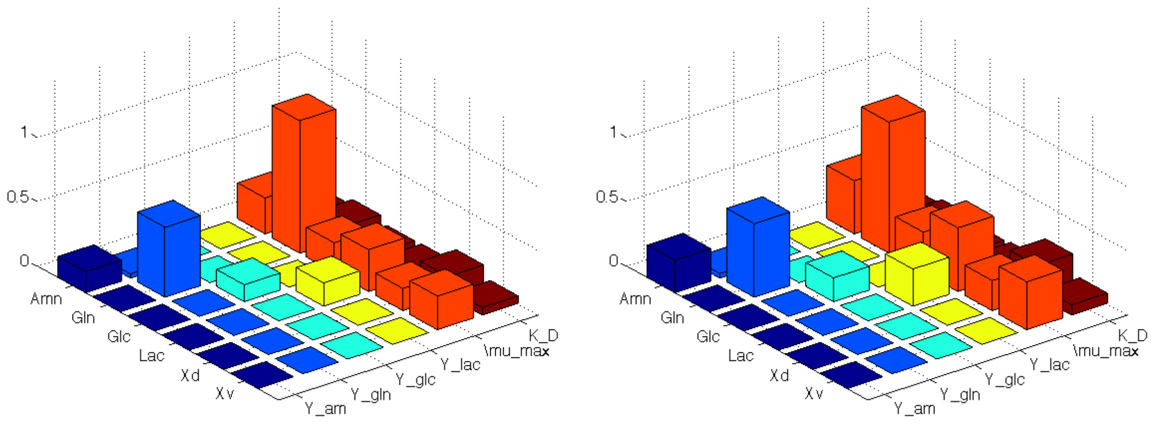

(a) local sensitivity coefficients $|S_{ij}|_\infty^{loc}$ (b) global sensitivity coefficients $|S_{ij}|_\infty^{glob}$

Figure S1. Parameter sensitivity analysis results for the exponential growth phase.

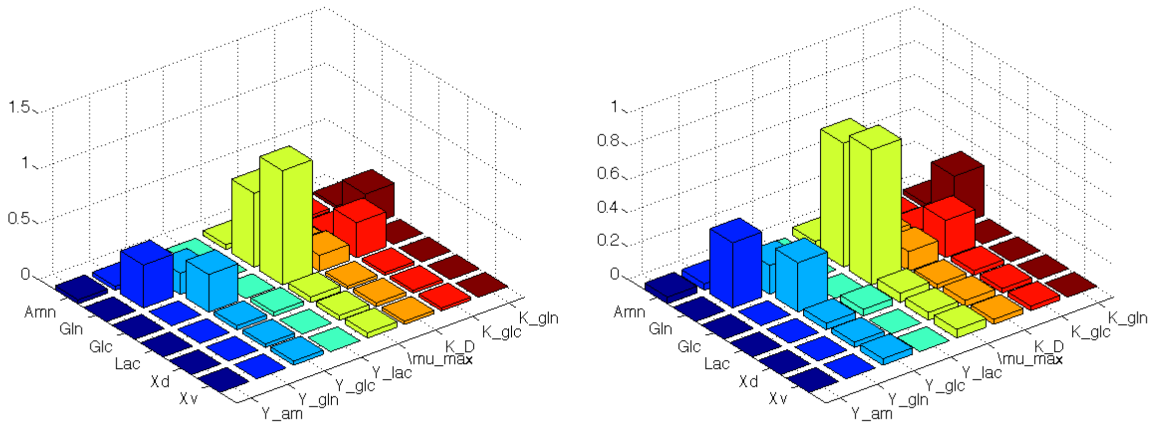

(a) local sensitivity coefficients $|S_{ij}|_\infty^{loc}$ (b) global sensitivity coefficients $|S_{ij}|_\infty^{glob}$

Figure S2. Parameter sensitivity analysis results for the complete time course of the measurements.

Monod constants K_{Glc} and K_{Gln} are shown sensitive. Furthermore, the relative influence of changes in the product yield factors $\mathrm{Y}'_{\mathrm{X/Amn}}$ and $\mathrm{Y}'_{\mathrm{X/Lac}}$ is smaller than in the exponential growth phase.

Though qualitatively the results obtained through PSA are consistent with the proposed sensitivity measure, a comparison of the methods is complicated. Local/global PSA has the advantage that the influence of infinitesimal small parameter changes with respect to particular species can be evaluated. However, interpretation of PSA results is in general very difficult, e.g. the results can not be extrapolated to evaluate the effect of significant variations in the parameters e.g. due to inherent variability. The proposed sensitivity measure on the contrary directly indicates those parameter variations such that the model is still acceptable.

Outlier Analysis

In this section, we cross-validate the identified outliers considering the z-score, Grubbs test for outliers [3] if applicable and otherwise the trimmed sum of squares (LTS), see [4].

z-score The z-score of an observation is defined for normally distributed errors as

$$z = \frac{\tilde{x} - \overline{x}}{\sigma},$$

where $\tilde{x}$ denotes the observation, $\overline{x}$ the sample mean, and σ the standard deviation of the errors. In the present case, we know from assay validation that the measurement errors of the species *Amn*, *Glc*, and *Lac* are normally distributed (and unbiased). The z-score is thus given by

$$z = \frac{\tilde{x}_i(t_j) - \overline{x}_i(t_j)}{\sigma_i}, \tag{5}$$

where $\tilde{x}_i(t_j)$ denotes the observation of species x_i at t_j and σ_i the standard deviation according to Table 1. For the species *Gln*, X_d, and X_v a relative error r_i (see Table 1) is considered, therefore we set $\sigma_i = r_i \tilde{x}_i(t_j)$.

In Eq. 5, $\overline{x}_i(t_j)$ denotes the model prediction of x_i at t_j considering the parameters obtained from a standard least squares method (using Simbiology toolbox [5]). We distinguish two predictions: First a prediction based on the parameter obtained *without* removing the suspected outlier (denoted by $\overline{x}_i(t_j)|_{prior}$), and second based on the parameter obtained after removing the outlier ($\overline{x}_i(t_j)|_{post}$). This leads respectively to two z-scores, z_{prior} and z_{post} respectively.

Grubbs Test The Grubbs (single) outlier test is as follows: The hypothesis 'no outlier' is rejected at significance level α if

$$z > z_{crit} = \frac{N-1}{\sqrt{N}} \sqrt{\frac{(t_{\alpha/2N,N-1})^2}{N-2+(t_{\alpha/2N,N-1})^2}}, \tag{6}$$

see [3], where $t_{\alpha/2N,N-1}$ denotes the upper critical value of the t-distribution with $N-2$ degrees of freedom. We consider the standard significance level $\alpha = 0.05$, and for the bioreactor experiment, we have for each specie $N = 7$ observations (thus $z_{crit} = 2.02$), and for the shaker experiment $N = 14$ observations (thus $z_{crit} = 2.51$).

The Grubbs test is positive if the 'no outlier' hypothesis is rejected. The Grubbs test before deletion of the outlier is denoted by GT_{prior} considering z_{prior}, after deletion GT_{post} considering z_{post}.

Least trimmed squares (LTS) The LTS is a modification of the standard least squares method. The least trimmed squares method minimizes the sum of squared residuals over a subset of observations, for details refer to [4]. In the present case, we focus on single outlier hypothesis only, i.e. utilizing $N-1$ observations. We use LTS to find the $N-1$ observations that yield the lowest sum of squared residuals. Conversely, this means to find the single observation whose removal is optimal in LTS sense and thus an outlier candidate. For evaluation, we again utilize the Simbiology toolbox [5].

We say the LTS test is positive, if the LTS is minimal for the (suspected) outlier regarding any other possible single outlier hypothesis.

Table S1. Outlier validation results: Bioreactor

**outlier**	**before deletion**		**after deletion**		**LTS**
	z_{prior}	GT_{prior}	z_{post}	GT_{post}	
$X_d(43.5\ h)$	2.5	+	4.0	+	+
Lac(137.3 h)	5.6	+	7.6	+	+
Amn(137.3 h)	20.2	+	20.3	+	+

Table S2. Outlier validation results: Shaker

**outlier**	**before deletion**		**after deletion**		**LTS**
	z_{prior}	GT_{prior}	z_{post}	GT_{post}	
Gln(0 h)	0.9	-	1.24	-	+
$X_v(69.2\ h)$	1.6	-	2.2	-	+
$X_d(47.0\ h)$	11.1	+	11.3	+	-
$X_d(152\ h)$	1.4	-	1.5	-	+
Lac(x^*)	≥3.8	+	≥6.0	+	

*x=$\{34\ h, 47\ h, 54.8\ h, 69.2\ h, 81.5\ h\}$.

Results

We apply above tests to consolidate the outlying observation identified using our method. Recall that we utilized the 1-σ confidence intervals for the measurements as hard constraints, and the outliers have to be understood in this sense.

The results for the bioreactor are given in Table S1, and for the shaker experiment in Table S2. All outlier candidates in the bioreactor are validated considering these additional tests. For shaker experiment, the tests showed that $X_d(47.0\ h)$ can be safely considered as an outlier. Although LTS test is positive for $Gln(0\ h)$, $X_v(69.2\ h)$, and $X_d(152\ h)$, their deviation may also be explained by natural deviation. Regarding the consecutive outliers in the lactate dynamics, a model mismatch is the most appropriate explanation, e.g. due to pyruvate consumption which has however not been measured.

References

[1] Leis JR, Kramer MA (1988) The simultaneous solution and sensitivity analysis of systems described by ordinary differential equations. ACM Trans Math Softw 14: 45–60.

[2] Helton J, Davis F, Johnson J (2005) A comparison of uncertainty and sensitivity analysis results obtained with random and latin hypercube sampling. Reliability Engineering & System Safety 89: 305–330.

[3] Grubbs F (1969) Procedures for detecting outlying observations in samples. Technometrics 11: 1–21.

[4] Hawkins D (1994) The feasible solution algorithm for least trimmed squares regression. Computational statistics & data analysis 17: 185–196.

[5] MATLAB (2012) Simbiology Toolbox 2. Natick, Massachusetts: The MathWorks Inc.
